# Intrapelvic Cup Migration Following Revision Total Hip Arthroplasty: A Case Report and Review of the Literature

**DOI:** 10.7759/cureus.51498

**Published:** 2024-01-02

**Authors:** Spyridon Papagiannis, George Sinos, Christiana Kotsia, Irini Tatani, Panagiotis Megas

**Affiliations:** 1 Orthopaedics and Traumatology, Patras University Hospital, Patras, GRC; 2 Radiology, Patras University Hospital, Patras, GRC

**Keywords:** total hip arthroplasty revision, revision, total hip arthroplasty, complication, cup protrusion, intrapelvic migration

## Abstract

Intrapelvic acetabular cup migration is a rare but serious complication that can occur following either primary or revision total hip arthroplasty. Medial acetabular wall weakening is considered the main predisposing factor for acetabular protrusion. A thorough preoperative plan is essential to advocate proper pelvic anatomy reconstruction, including osteosynthesis of the pelvis, if necessary, preservation of muscle and bone stock, and selection of the right prosthetic components without damaging adjacent anatomical structures. We present a rare case of an 84-year-old woman with a hip dislocation and complete intrapelvic migration of the acetabular component, nine years after her second revision surgery of a hip prosthesis placed 60 years ago due to congenital hip dysplasia. The protruded acetabulum was not removed since preoperative CT and digital subtraction angiography (DSA) revealed no vascular compromise. A non-cemented, tantalum acetabular cup, reinforced by a short flange titanium acetabular cage, was placed with a cemented, polyethylene-bearing surface, which was revised to a cemented, constrained acetabular insert three months postoperatively due to dislocation after mobilization on the bed. We conducted a literature review to elucidate the causes, proper diagnostic tools, and preoperative planning of this rare occurrence while trying to evaluate a potential treatment protocol.

## Introduction

Intrapelvic cup protrusion following total hip arthroplasty is defined as the migration of any prosthesis-related component at least 15 mm medial to the ilio-ischial (Köhler’s) line [1]. Although rare and not commonly reported in global registries among the causes leading to revision arthroplasty [2], it is considered extremely serious and is directly associated with potentially lethal complications such as neurovascular injuries [3] and damage to the intrapelvic organs [4]. Progressive mechanical loosening, implant malposition with chronic instability, injuries accompanied by acetabular fractures, and infection [5] can all compromise medial acetabular wall strength, leading to intrapelvic component migration. The protrusion can involve the entire prosthesis, the cup separately, cement only, or any metallic material; however, no specific classification system has been described. Paprosky classification for acetabular bone loss in revision hip arthroplasty defines class IIIB cases as superomedial, ‘up and in’, implant migration with disrupted Köhler’s line [6]. The French Society for Hip and Knee Surgery attempted to categorize the degree of protrusion in nine zones delineated by two horizontal lines, connecting the distal ends of the sacroiliac joints superiorly and the teardrops inferiorly, and by two vertical lines that ran along the ilio-ischial line and the sacrum-pubis axis, respectively. Protrusion to the lateral column was defined as type I, to the medial column as type II, and the central column as type III [1]. The purpose of this study is to present a rare case of complete intrapelvic cup migration following revision hip surgery, where the protruded component was left in situ.

## Case presentation

**Figure 1:**
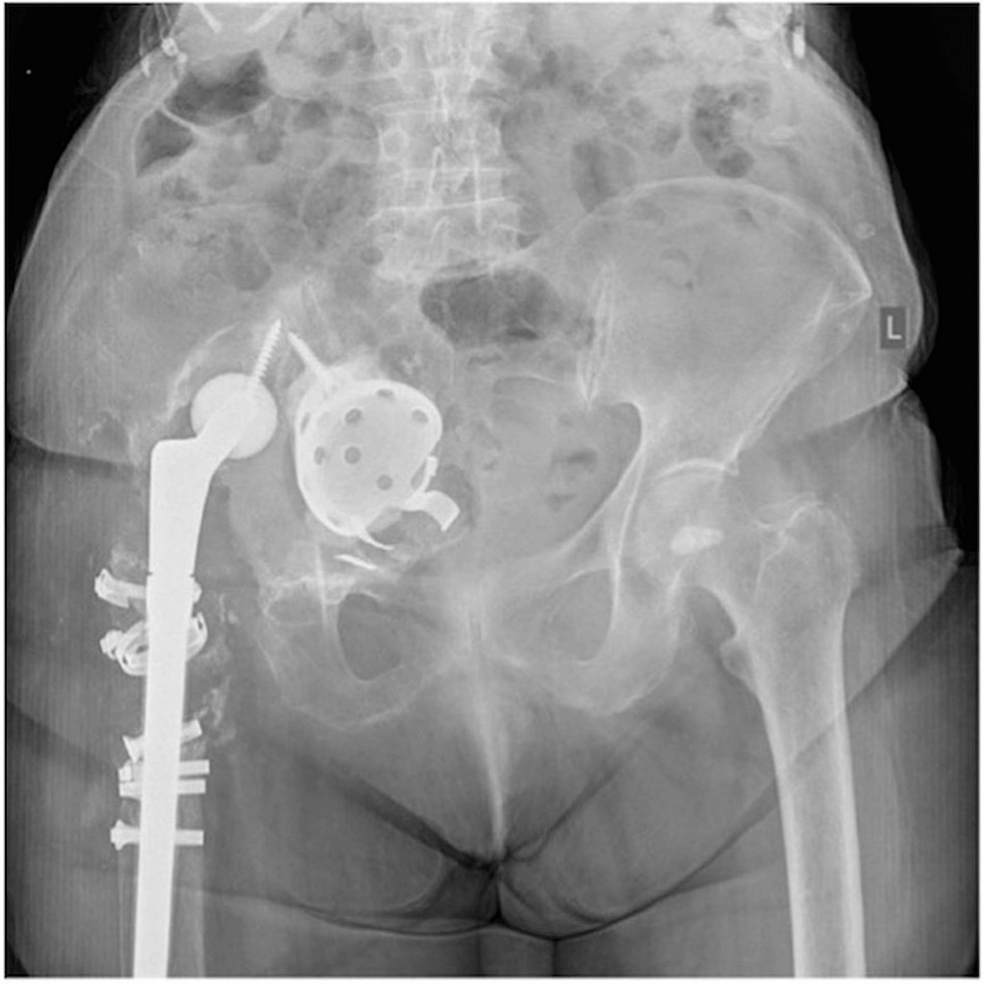
A preoperative radiograph shows complete intrapelvic cup protrusion, pelvic discontinuity with complete distortion of the medial acetabular wall, and excessive osteolysis and absorption of the iliac bone.

The patient, an 84-year-old female with a history of hypertension and atrial fibrillation, presented to the outpatient department of our clinic complaining about right hip pain and an inability to bear weight. Symptoms began about three months prior and progressively deteriorated, with no history of trauma. The patient's right limb was internally rotated and unable to perform any active movement, raising concerns for a potential dislocation, while a leg length discrepancy of 3 cm was identified. Neurovascular status was found to be intact. The patient had a history of right hip replacement at the age of 24 due to congenital hip dysplasia, which was revised 30 years later due to aseptic implant loosening. Twenty years after her first revision surgery, the patient underwent a second reoperation, again because of aseptic component loosening. Since then, she has been able to walk with assistance, while she described having persistent mild right hip pain. Radiographs revealed complete intrapelvic cup protrusion, with the acetabular component rotated inside the pelvis and migrated to the central column. Pelvic discontinuity was identified, with complete distortion of the medial acetabular wall and excessive osteolysis and absorption of the iliac bone. Extensive bone absorption was also present at the proximal femoral shaft region, while the shaft was fixed in the varus position (Figure 1).

**Figure 2:**
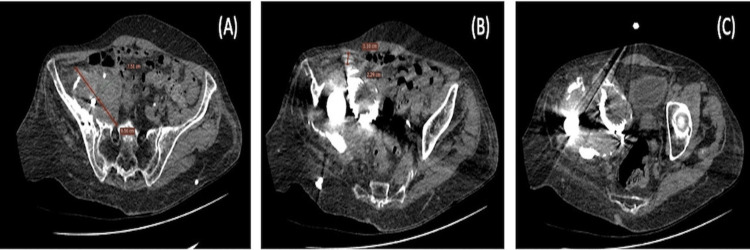
A non-enhanced axial CT showing a hyperdense mass-like lesion surrounding the protruded cup (A), lymph nodes surrounding the lesion (B), and significant displacement of the bowel, bladder, and uterus (C).

A CT scan was performed to evaluate potential vascular involvement, the proximity of the migrated component to intrapelvic anatomical structures, and better visualization and interpretation of pelvic discontinuity and available bone stock. The CT scan showed a hyperdense mass-like lesion surrounding the protruded cup in the right hemipelvis (Figure 2A).

**Figure 3:**
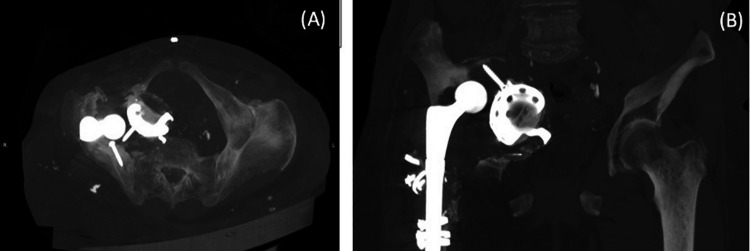
A 3D reconstruction demonstrating existing bone loss 3D: three-dimensional

Imaging differential considerations included metal-on-metal pseudotumor and myositis ossificans. Multiple hyperdense lymph nodes were surrounding the lesion, with maximum dimensions of 11x23 mm (Figure 2B). Consequently, a mass effect was identified, with significant displacement of the bowel, bladder, and uterus (Figure 2C). No apparent participation of the intra- or retroperitoneal organs or the visceral contents of the pelvis in the inflammatory process was shown. Three-dimensional (3D) reconstructions demonstrated the remodeling of the right innominate bone and the exact location of the protruded cup and two screws. Acetabular bone loss was classified as Paprosky type IV (Figures 3A, 3B).

**Figure 4:**
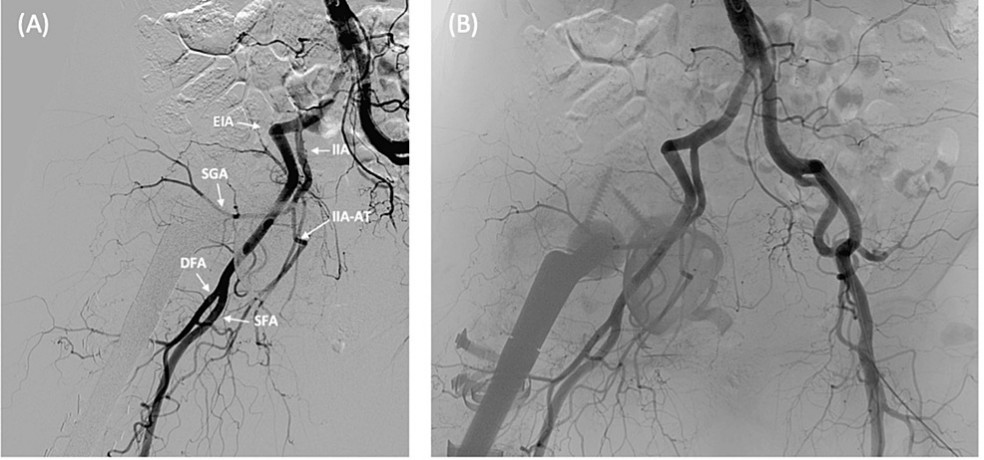
Digital subtraction angiography demonstrated no arterial injuries to the external and internal iliac arteries (EIA, IIA), superior gluteal artery (SGA), the anterior trunk of the internal iliac artery (IIA-AT), as well as the superficial and deep femoral arteries (SFA, DFA).

Digital subtraction angiography (DSA) confirmed the absence of any vascular injuries (Figures 4A, 4B).

**Figure 5:**
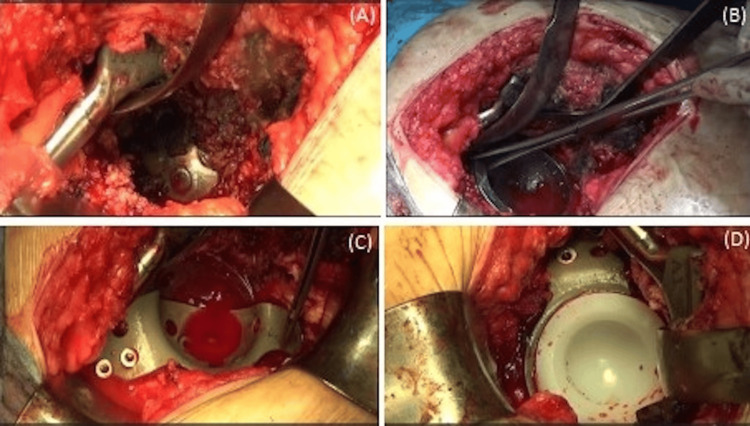
Intraoperative images demonstrate the protruded cup left in situ (A), a well-fixed tantalum cup (B) reinforced by a short, short-flange titanium acetabular cage (C), and the placement of a cemented polyethylene-bearing surface (D).

Laboratory studies, including white blood cell count (WBCs), erythrocyte sedimentation rate (ESR), C-reactive protein (CRP), and blood cultures, were normal, excluding potential infection. The patient was admitted to the orthopedic ward, and a surgical intervention was scheduled. In the absence of a neurovascular or other intrapelvic organ injury, a decision was made not to remove the protruded cup since it could seriously increase the danger of iatrogenic damage. With the patient in the lateral decubitus position under general anesthesia, a posterior approach using the previous skin incision was utilized. The acetabulum was debrided and reamed until contact was made with the bleeding host bone. The defects were filled with morselized bone allografts using the reverse-reaming technique. A 66-mm-diameter tantalum revision shell (Zimmer Biomet®, Warsaw, IN) was placed within the acetabulum and fixed with two screws. The tantalum cup was then reinforced by the short flange titanium acetabular cage 66/68/70 (Zimmer Biomet®). The inferior fixation of the cage was obtained by impacting the ischial flange into the ischium. The superior flange was stabilized proximally to the iliac bone remnants with three screws. A cemented polyethylene-bearing surface with a diameter of 32 mm was placed (Figures 5A-5D).

**Figure 6:**
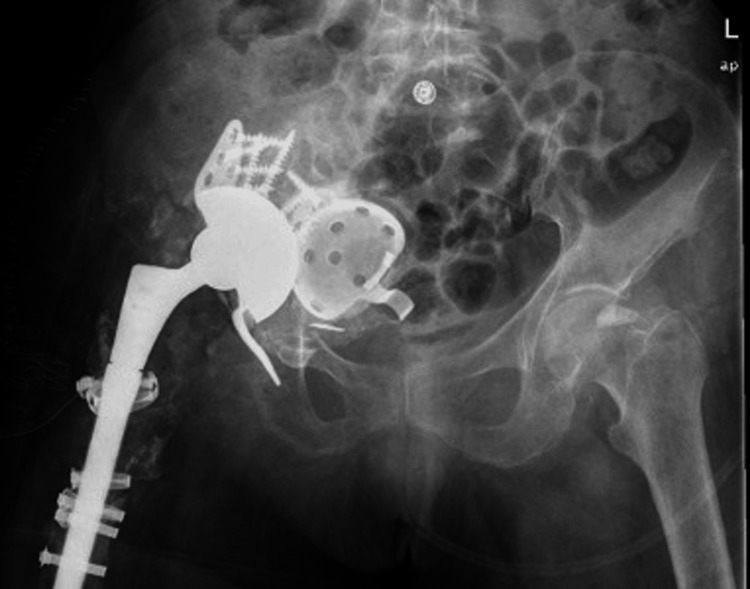
Postoperative radiograph following acetabular reconstruction while maintaining the protruded cup.

The femoral shaft component was found to be stable intraoperatively, thus deciding not to replace it in order to avoid further blood loss and prolongation of operation time. A 32-mm-diameter, metallic head was utilized, and the hip was relocated. No signs of instability were identified, a draining tube was placed, and the incision was closed in the usual manner (Figure 6).

**Figure 7:**
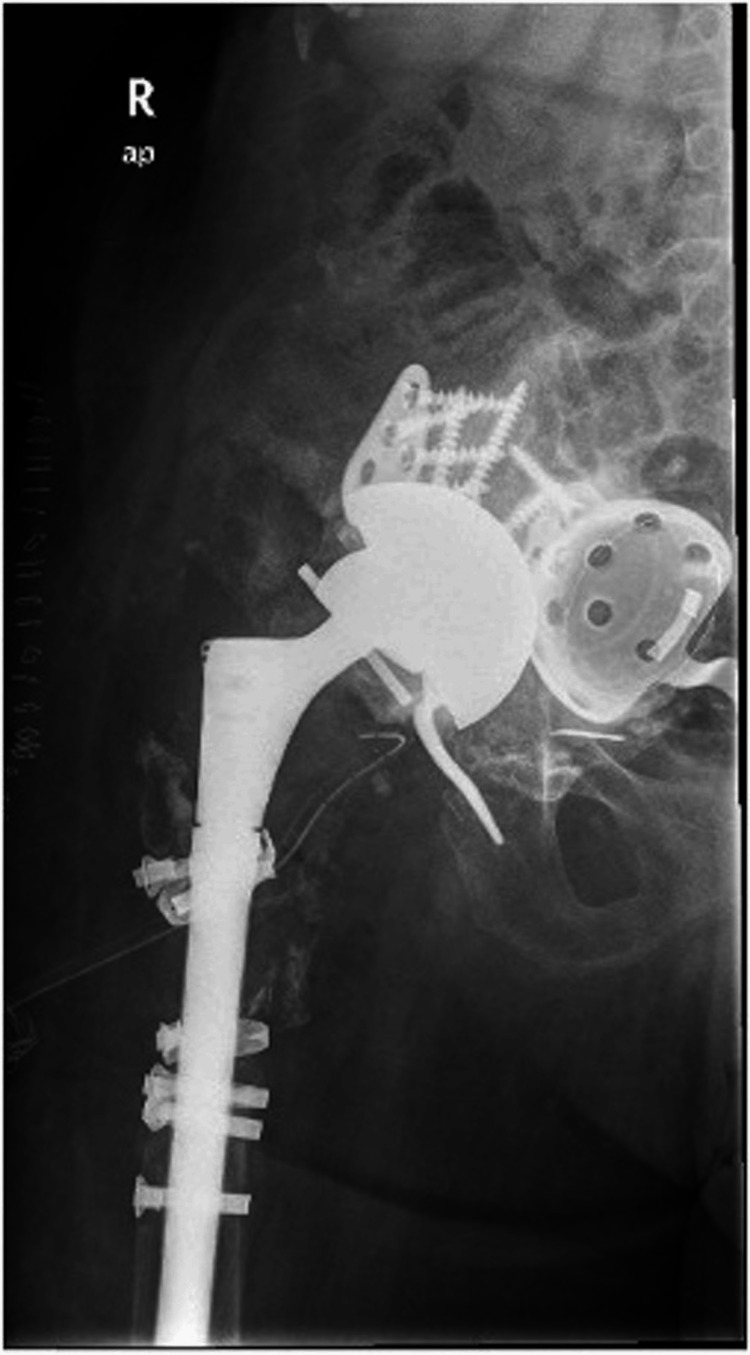
Postoperative radiograph after the placement of a constrained polyethylene inlet.

Six days postoperatively, during mobilization on the bed, the patient complained about acute right hip pain, while the patient’s lower extremity was in abduction and external rotation. A postoperative dislocation was confirmed by plane radiographs, and the patient underwent a closed reduction under general anesthesia on the same day. During a prolonged hospitalization of three months in total due to a complicated, COVID-related respiratory tract infection, the patient was unable to bear weight while describing a recurrent sense of instability and ‘giving way’ of her right hip even in slight on-bed movements. The decision was made to revise the previous surgery using a constrained acetabular insert (Trident® Constrained Acetabular Insert, Stryker®, Kalamazoo, MI) this time (Figure 7).

Postoperatively, the patient was able to walk with assistance, and after an uncomplicated hospitalization of 10 days, she was discharged. After a follow-up period of 12 months, the patient was able to bear weight using crutches; no episodes of instability were mentioned, and a Harris hip score (HHS) of 80 was documented.

## Discussion

Intrapelvic cup migration appears to be uncommon, but its frequency is beginning to increase as more prosthetic devices are implanted, life expectancy rises, and mechanical loosening events occur more frequently after hip replacement surgeries. A protrusion is typically a progressive phenomenon. As a result, a thick capsule, of almost 1 cm, may develop around intrapelvic protruding components, thus providing some protection to the noble adjacent structures. This mechanism may explain the low total incidence of intrapelvic injuries after failed total hip arthroplasty [7]. The medial acetabular wall is mostly compromised in cases of severe migration, exposing intrapelvic structures. The level at which the acetabular wall has been breached as well as the orientation of intruding components are directly associated with the complication risk [8]. In a meta-analysis published by Bach et al. regarding intrapelvic complications following hip arthroplasty, five major categories were identified, namely, intrapelvic nerve and vessel complications, urogenital and intestinal tract complications, and intrapelvic mass formation [9]. Structures mostly at risk include the external iliac artery, the bladder, the sigmoid colon, the sciatic nerve, and the iliopsoas muscle. Among vascular injuries, false aneurysms are the most prevalent. Ongoing compression and erosion of the vessel wall may cause laceration with continuous leakage of blood into the surrounding tissues, thus leading to pseudoaneurysm formation [10]. Fistula formation is the most frequent complication associated with both the urogenital and intestinal tracts, while nerve injuries are commonly due to compression. In general, complications commonly emerge due to intrapelvic cement extension rather than intruding acetabular components.

Clinical examination and imaging evaluation are key elements in the preoperative assessment of patients with protruded intrapelvic components. Signs of neurological deficit and/or irritation, in particular, lumbosacral plexus compression in cases of components being adjacent to the sacroiliac joint, in addition to signs of digestive, urinary, and/or gynecological function, should be interpreted. Anteroposterior (AP) pelvic and hip X-rays, lateral hip images, and three-quarter views can be used to evaluate pelvic continuity. Computerized tomography imaging can also be useful to distinguish pelvic discontinuities and areas of bone loss, as well as potential acetabular wall fractures. However, imaging quality can be commonly compromised by artifacts. Computed tomography angiography (CTA), including the late venous phase, although not performed in our case, is the primary supplementary exam, showing vascular components and their relationship with nerves, urogenital, and digestive structures. A CTA is considered superior to DSA, which can provide neither information about the distance between migrated implants and adjacent structures nor sufficient mapping due to the superimposition of various anatomical structures. A full blood workup should also be included to exclude potential infections [11].

Few data are available describing this rare entity, mostly emerging from case reports and limited case series (Table 1).

**Table 1:** Cases of intrapelvic cup migration THA: total hip arthroplasty; CT: computer tomography; ORIF: open reduction internal fixation; CTA: computer tomography angiography; N/A: not available

Authors	Background	Preoperative imaging	Approach	Reconstruction method	Outcomes
Murcia-Asensio et al. [12]	Female, 70 years old. THA for subcapital fracture. Acute migration. No reoperations.	CT and X-rays. Comminuted posterior wall fracture with displaced lamina quadrilateral. No vascular imaging.	Combined Stoppa and posterolateral approach.	Implant removal. ORIF of lamina quadrilateral with a Matta plate. ORIF of the hemi-transverse acetabulum fracture with a reconstruction plate. Morselized bone allograft was impacted on a flexible medial wall metal mesh to manage bone defects. A press-fit Delta cup reinforced with two screws and a dual mobility metal insert was set in place.	Six-month follow-up. The patient returned to the same level of activity.
Fenelon et al. [13]	Male, 65 years old. Chronic, septic loosening occurred 19 years following primary arthroplasty. Three failed two-stage revisions.	Angio-CT: distortion of the bladder and right ureter with compression of the external iliac vein and artery.	Combined paramedial abdominal approach and lateral approach with trochanter osteotomy.	Insertion of a right ureteric stent to provide protection and facilitate identification of the right ureter. A meticulous dissection of vessels from the fibrous, inflammatory capsule. Acetabular component removal.	Intravenous outpatient antibiotic therapy. Three-month follow-up. Mobilization using crutches. Wound healing.
Chana-Rodríguez et al. [14]	Male, 71 years old. THA was performed 10 years ago; revision was done five years ago due to aseptic loosening. Chronic aseptic migration.	CTA and X-rays. No obvious vascular defect.	Combined Stoppa and posterolateral approach.	Implant removal through the Stoppa approach. Acetabular mesh with morselized cancellous bone allograft implantation to reinforce deficient bone stock. Trabecular metal revision cup implantation with an additional cage reinforcement cemented polyethylene liner.	10-month follow-up. Painless, non-assisted weight bearing.
Grigoris et al. [15]	Nine cases: eight males and one female. 64 years on average. 13 years from primary THA on average. Chronic aseptic migration. One case with a history of reoperation.	X-rays. CTA was used in five cases.	Combination of two approaches: incision from the anterior half of the iliac crest to the midpoint of the inguinal ligament. Lateral incision in five cases. Anterior extension in four cases.	Protruded components were removed through the ilioinguinal approach. A variety of prostheses and bone grafting were used for acetabular reconstruction.	N/A
Abdelnasser et al. [16]	Male, 63 years old. THA for avascular necrosis seven years ago. Chronic aseptic protrusion. No reoperations.	CTA, X-rays. Extensive osteolytic defect with suspicion of pelvic discontinuity and mild displacement of the external iliac vessels.	Combination of pararectus and a direct lateral approach.	The external iliac artery and vein, iliacus, and psoas muscles with the femoral nerve were isolated and retracted. Cup extraction was followed by anterior column ORIF to restore pelvic continuity.	N/A
Salvi et al. [17]	Two cases of protruded, verticalized cups: a cemented one and a non-cemented one.	N/A	N/A	N/A	N/A
Sciberras et al. [18]	Female, 78y; revision THA; chronic aseptic protrusion.	CTA, X-rays. Loose revision hip replacement, deficient medial acetabular wall, and intrapelvic migration. No relationship to intrapelvic vessels	Hardinge's approach through the previous scar was used and extended up to the iliac crest.	The acetabulum was cleared down to the teardrop, and the pseudomembrane was removed. Two trabecular metal augments were used to snugly fill the superomedial defect. The remaining defect was filled with a morselized bone graft. The revision shell was inserted and held with multiple self-tapping screws. An acetabular revision cage system with a short flange was chosen. The cage was then inserted into the acetabular shell and secured in place with cancellous screws. The acetabular cup was cemented into an anatomical position.	There were no hip-related problems after a follow-up period of 30 months.

Moreover, reports on preoperative evaluation, prognostic factors, surgical techniques, and outcomes in patients undergoing revision arthroplasty after intrapelvic cup migration are even scarcer in recently published literature. A review article published by Epinette et al. reported a total of 246 cases of intrapelvic cup protrusion with a minimum follow-up of two years [1]. The mean age at cup revision was 66 years; the time since failed arthroplasty with protrusion was longer than 10 years on average; and a history of previous cup revision was noted in 50% of the cases, including 12% with three or more previous operations, like ours. Aseptic loosening was the primary cause in 93% of hips. Standard radiographs were the main diagnostic tool, while vascular imaging was performed in only 42 (17%) cases. A wide range of approaches were utilized, with typical posterolateral and anterior approaches being used in almost 90% of the cases. Uncemented cup fixation was predominately chosen, using allograft/autograft or bone substitute techniques to manage bone loss in 91% of the cases and metallic reconstruction devices in 154 (62% of cases) to enhance acetabular stability. The mean Postel-Merle d'Aubigné (PMA) score improved from 8.7 ± 2.4 preoperatively to 14.2 ± 3.1, achieving a “forgotten hip” status (PMA score of 18 points) in 17% of cases. The mean HHS improved from 42.2 ± 16.4 to 78.0 ± 18.7 postoperatively. Complication rates were 29%, including dislocation (8.5%), vascular (7.1%), or sciatic nerve (1.96%) injuries, as well as implant rupture (1.96%). Cup revision was required in 40 (16.3%) cases due to instability/dislocation (eight cases), infection (eight cases), and aseptic loosening (24 cases), with a recurrent loosening time shorter than 10 years in 96% of the cases [1].

Salvi et al. [17] were the first to report two cases of verticalized, protruded acetabular cups, a cemented and a non-cemented one, that were left in place during revision surgery. According to the authors, it is possible to leave a migrated but well-fixed cup in place simply by loading it through the insertion of a second cemented cup, thus minimizing surgical time and blood loss. The unremoved prosthetic cup, which is better stabilized by the addition of the new cemented component, can work as a "supporting wall" in a technique described as the ‘’wall-socket’’ technique. Sciberras et al. [18] described a case of a 78-year-old woman with medial acetabular wall deficiency and intrapelvic cup migration following revision total hip arthroplasty where the protruded cup was left in situ. A CTA revealed no close relationship to significant intrapelvic vessels, thus deciding to retain the protruded cup.

To our knowledge, this is the fourth reported case with complete intrapelvic cup migration where the protruded cup was not retrieved. Although the CT scan described the lesion surrounding the protruded cup and the resulting mass effect on the intrapelvic structures, no signs of vascular injury were confirmed. A posterior approach was utilized according to the surgeon’s preference, providing sufficient visualization of the acetabulum. An extensive medial acetabular defect was recognized, while the protruded cup was visible and palpable through the deficient acetabular wall. The fibrous cup capsule was intact, and further surgical manipulations in proximity to the cup were avoided to minimize the risk of iatrogenic vascular or nerve injury due to the distorted anatomy of the area. However, no interference of the intrapelvic cup with acetabular reaming or tantalum cup fixation was observed intraoperatively. The short-flange titanium acetabular cage and the cemented bearing surface were well-positioned, while no signs of instability were identified. However, acute postoperative dislocation and recurrent instability presented as the most common complications presenting after such complex procedures. Instability was probably caused by a less anteverted cup, given the difficulties of completely understanding proper prosthesis positioning and orientation in a situation with completely distorted anatomical landmarks. A constrained cup was selected during reoperation based on the patient’s age and low postoperative demands. Finally, after a follow-up period of 12 months, the patient was able to walk with assistance, experiencing minor hip pain while being completely independent.

## Conclusions

Intrapelvic cup migration remains an extremely rare complication following primary and revision total hip replacement, which is associated with neurovascular injuries and damage to adjacent intrapelvic structures. A thorough clinical examination, radiographs, and CTA are considered the main tools for diagnosis and proper preoperative planning. A multidisciplinary approach in collaboration with surgeons from radiology, general, vascular, and urology departments may be required to understand the correlation of the protruded cup to the intrapelvic organs and to facilitate the proper treatment. Meticulous attention during surgical management of this challenging condition is of great importance, as is being able to utilize different approaches and a large variety of surgical instruments and implants to restore normal pelvic anatomy.
